# Partnered Pharmacist Medication Charting for Children and Young People: A Retrospective Cohort Study

**DOI:** 10.1111/jpc.70505

**Published:** 2026-07-28

**Authors:** Georgia Lloyd, Brooke McCarthy, Polly Ng, Christopher Johansson, Felicity Wright

**Affiliations:** ^1^ Sydney Children's Hospital, Randwick Sydney New South Wales Australia; ^2^ School of Health Sciences University of New South Wales Sydney New South Wales Australia; ^3^ School of Pharmacy University of Sydney Sydney New South Wales Australia

**Keywords:** emergency department, medication errors, paediatrics, partnered pharmacist medication charting, pharmacist prescribing, transitions of care

## Abstract

**Aim:**

Partnered pharmacist medication charting (PPMC) reduces medication errors in Australian adult hospitals. This study evaluated the impact of PPMC on paediatric emergency department (ED) presentations compared to early best possible medication history (BPMH) and usual care.

**Methods:**

A retrospective cohort study was conducted at a tertiary paediatric hospital with electronic medication management systems, comparing three sequential models of care across three time periods: usual care (T1), early BPMH by an ED pharmacist (T2), and PPMC (T3). The primary outcome was the proportion of patients with one or more medication errors, assessed for clinical significance by a blinded expert panel. Secondary outcomes included length of stay (LOS) and medication‐related documentation completion.

**Results:**

The percentage of patients with one or more medication errors identified was 70.6% in usual care (T1, *n* = 126), 50.8% in early BPMH (T2, *n* = 122), and 5.8% in PPMC (T3, *n* = 103). The absolute risk reduction for T3 PPMC compared to T1 Usual care (ARR T1 vs. T3) was 0.65 (95% CI 0.558–0.742) with one medication error prevented for every two patients receiving PPMC (NNT = 2, 95% CI 2–2). The median LOS was 3.3 days in usual care (T1) and 2.3 days in PPMC (T3) (*p* = 0.012).

**Conclusions:**

PPMC substantially reduced medication errors and the prevalence of high or extreme risk errors. The benefit extended beyond error prevention, with improvements in medication reconciliation, dosing weight documentation, and allergy review, and an association with shorter LOS. These findings support PPMC as a scalable approach to medication safety in paediatric hospitals.

## Summary

- What's known on this subject?
  ○ Partnered pharmacist medication charting (PPMC) is a collaborative prescribing model of care used in Australian adult hospitals to mitigate preventable medication‐related harm and enhance patient flow within health systems.
  ○ PPMC has demonstrated improvements in patient safety, is associated with a reduced length of stay, and cost savings per adult patient admission.
  ○ Children and young people are more likely to experience medication errors than adults. Evidence for PPMC is limited to adult cohorts and has largely been explored in paper‐based settings.
- What this study adds?
  ○ This retrospective cohort study evaluates PPMC in patients younger than 19 years presenting to a hospital emergency department (ED) with electronic medication management systems.
  ○ This study demonstrates that PPMC significantly reduces medication errors and improves medication‐related documentation in children and young people presenting to the ED compared to usual care and an early best possible medication history.
  ○ An association with a reduced length of stay (LOS) was observed with PPMC compared to usual care, though causality cannot be inferred from this retrospective design.

## 1 Introduction

Medicines are the most commonly used healthcare intervention and a leading source of preventable patient harm [1]. Medication errors are among the most frequently reported clinical incidents [1], defined as “any preventable event that may cause or lead to inappropriate medication use or patient harm while the medication is in the control of the health care professional, patient, or consumer” [2]. Errors occur across prescribing, dispensing, administration, and monitoring [3], with 53% occurring during prescribing [4].

Paediatric patients experience approximately three times the rate of potential adverse drug events (ADEs) compared with adults, driven by factors including weight‐based dosing, variable pharmacokinetics, and limited ability to communicate symptoms [5, 6]. It should be noted that Kaushal et al. measured potential ADEs and medication error preventability rather than a direct measure of harm. Error prevalence ranges from 10% to 15%, with dosing errors being the most common, accounting for 39% to 49% of reported errors [6].

The 2017 World Health Organisation (WHO) Global Patient Safety Challenge: Medication without Harm aimed to halve preventable medication‐related harm within 5 years, highlighting transitions of care as a priority area [7]. Transitions of care carry high risk [8] with 22%–72.3% of paediatric patients found to have one or more medication errors on admission to hospital [9].

Partnered pharmacist medication charting (PPMC) is a collaborative prescribing model in which a pharmacist and medical officer jointly develop and co‐sign a medication plan that is charted by the pharmacist and communicated to nursing staff and families [10]. PPMC improves medication safety, is associated with a reduced length of stay (LOS) and associated costs in adult populations, and is now standard practice in most Australian states [10, 11]. Pharmacist prescribing authority, co‐signature requirements, and scope of charting vary across jurisdictions, shaped by state‐based legislation and local clinical governance.

Across Australian adult PPMC studies, error severity potential has been assessed with the Society of Hospital Pharmacists Australia (SHPA) risk classification consequence–probability matrix [12]. Random samples of errors identified at baseline showed high‐risk errors in 14.7% to 18.9% and extreme‐risk errors in 0.3%–0.7% of cases across rural/regional and oncology cohorts [13, 14].

Tasmanian researchers evaluated a three‐arm model (usual care, early best possible medication history [BPMH] and PPMC) in a paper‐based prescribing setting, finding that while early BPMH improved medication charting accuracy, PPMC was most effective in reducing errors [15]. High‐ and extreme‐risk errors were combined for analysis, with the proportion of patients experiencing at least one such error falling from 26.6% in usual care to 23.2% with early BPMH and 1.3% with PPMC [15]. However, evidence in paediatric settings and electronic medication management systems (eMMS) remains limited.

This study aimed to evaluate the impact of PPMC on presentation to a paediatric hospital compared with early BPMH and usual care, assessing medication error rate, type and potential severity, LOS, and medication‐related documentation.

## 2 Method

Pharmacists were credentialed to complete PPMC, requiring 2 years of full‐time hospital pharmacy experience with at least 6 months in paediatrics. Competency was assessed via a Clinical Competency Achievement Tool, Mini Clinical Evaluation Exercise, supervised Entrustable Professional Activities (Level 4‐ Independent practice) [16], and an observed structured clinical examination (OSCE) conducted by a Senior Pharmacist and Emergency Staff Specialist.

This study was conducted across three 12‐month time periods between September 2022 and October 2025. Eligible patients were retrospectively identified during three time periods: T1 (18 September 2022–18 September 2023), T2 (18 September 2023–18 September 2024), and T3 (9 October 2024–9 October 2025). In T1, patients received usual care; in T2, an early emergency department (ED) pharmacist BPMH; and in T3, PPMC on presentation (Figure 1).

**FIGURE 1:**
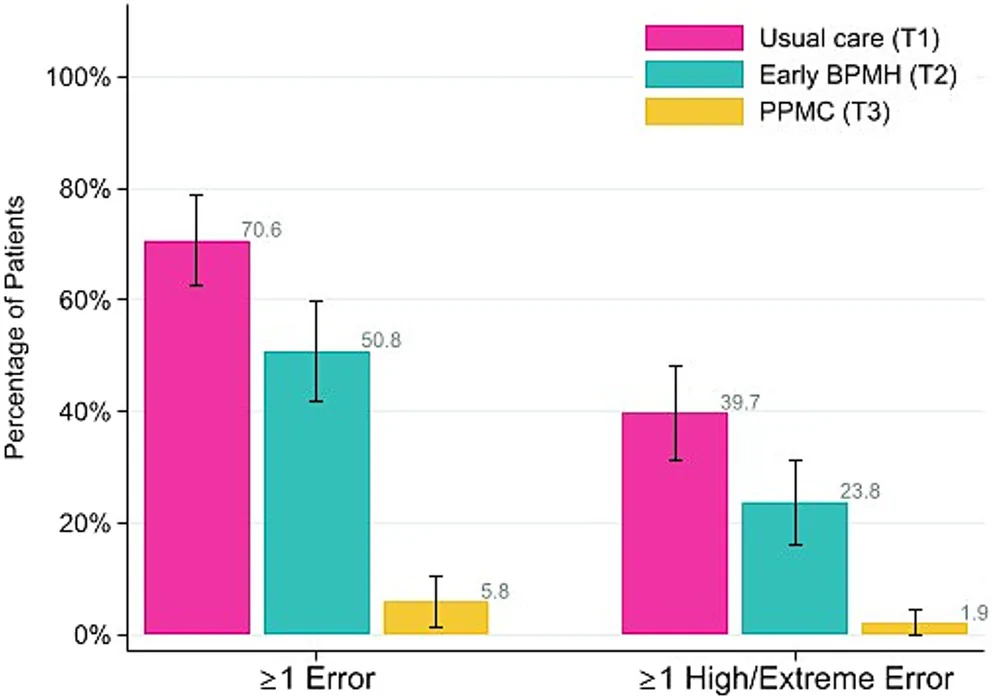
Proportion of patients with one or more medication error(s) and one or more high or extreme risk error(s), by model of care. Error bars represent 95% confidence intervals. T1 = Usual care, T2 = early Best Possible Medication History, T3 = Partnered Pharmacist Medication Charting.

Inclusion criteria were patients under 19 years of age presenting via the ED during pharmacist working hours (8:00–18:00), taking at least one regular home medication and with a BPMH taken by a pharmacist. Patients were excluded if PPMC was performed during credentialing or for emergency medications only, or if admitted during eMR downtime.

The primary outcome was the proportion of patients with one or more medication errors on presentation, defined as undocumented discrepancies between the BPMH and inpatient medication orders without documented rationale. Secondary outcomes were LOS and medication‐related documentation completion. Dosing weight was defined as the weight entered into the eMMS for weight‐based dose calculations, distinct from the nursing recorded measured weight. Haematology/oncology patients were excluded from this analysis due to chemotherapy‐specific dosing protocols. Data were collected by four trained data collectors and verified by a single senior investigator.

A blinded expert panel comprising a paediatric emergency medicine staff specialist and a senior paediatric pharmacist assessed the clinical significance of identified errors using de‐identified patient summaries, blinded to group allocation, estimating potential clinical outcome if the error persisted for 48 h. Errors were stratified using the SHPA risk classification consequence–probability matrix (severity 1–5: insignificant to extreme) [12]. Discrepancies were not adjudicated as intentional or unintentional due to resource and retrospective design constraints, consistent with prior Victorian PPMC studies [14]. Disagreements were resolved by consensus.

For documentation outcomes, absence of eMR documentation was treated as a negative outcome rather than missing data. Cases were excluded only where source documentation was inaccessible.

The authors declare that the research presented in this manuscript adheres to the ethical principles outlined by the Sydney Children's Hospitals Network Human Research Ethics Committee (HREC). All procedures involving human participants were conducted in accordance with the ethical standards of the Sydney Children's Hospitals Network, New South Wales Health, and the Declaration of Helsinki (1964), as revised in 2013. This study was approved by Sydney Children's Hospital Network HREC (Reference number: 2024/ETH01235). No external funding was received.

### 2.1 Statistics

Statistical analyses used Stata v17 (StataCorp, TX, USA). Proportional data were compared using Pearson's chi‐square with post hoc Marascuilo pairwise comparisons where significant. Continuous variables were assessed for normality using Shapiro–Wilk; non‐normally distributed data are reported as median (IQR) and compared using Kruskal–Wallis with Bonferroni‐adjusted Mann–Whitney *U*‐tests. ARR and NNT were calculated using GraphPad Prism 10.6.1 (Windows, Boston MA).

## 3 Results

### 3.1 Patient Characteristics

Between September 2022 and October 2025, 116 327 patients presented via the ED. Of these, 501 had a weekday pharmacist medication history. After excluding patients with no home medications (*n* = 140), post‐discharge BPMH entry (*n* = 7), eMR downtime (*n* = 1), emergency charting (*n* = 1), or training PPMC (*n* = 1), the final cohort comprised 351 encounters: T1 *n* = 126, T2 *n* = 122, and T3 *n* = 103.

Baseline demographics were largely comparable (Table 1). Age distribution did not differ significantly (*p* = 0.435). Female sex was more prevalent in T1 (58.7%) than T3 (41.7%; *p* = 0.030), with Marascuilo post hoc testing confirming a significant T1–T3 difference. Triage category was similar across groups (*p* = 0.400). The proportion of patients admitted under haematology/oncology was comparable between T1 and T3 but was significantly lower in T2 (19.7% vs. 40.5% and 35.9%, respectively; *p* = 0.001).

**TABLE 1:** Demographic characteristics.

Variables	Study group			
	Usual care (T1) (*N* = 126)	Early BPMH (T2) (*N* = 122)	PPMC (T3) (*N* = 103)	*p*
Age (years), *n* (%)				
< 1	11 (9%)	12 (10%)	7 (7%)	0.435
1–5	37 (29%)	33 (27%)	20 (19%)	
5–10	28 (22%)	34 (28%)	27 (26%)	
> 10	50 (40%)	43 (35%)	49 (48%)	
Sex, female, *n* (%)	74 (58.7%)	67 (54.9%)	43 (41.7%)	0.030^a^
Triage category, *n* (%)				
High acuity (1, 2)	61 (48%)	53 (43%)	54 (52%)	0.400
Low acuity (3, 4, 5)	65 (52%)	69 (57%)	49 (48%)	
Haematology or oncology, *n* (%)	51 (40.5%)	24 (19.7%)	37 (35.9%)	0.001^b^
Total number of home medications (Median [IQR])	3 [2, 6]	3 [2, 6]	6 [4, 10]	< 0.001^c^

Abbreviations: BPMH, best possible medication history; IQR, interquartile range; PPMC, partnered pharmacist medication charting.

^a^ Marascuilo pairwise comparisons for sex: Usual care vs. Early BPMH—not significant; Usual care vs. PPMC—significant; Early BPMH vs. PPMC—not significant.

^b^ Marascuilo pairwise comparisons for haematology or oncology status: Usual care vs. Early BPMH—significant; Usual care vs. PPMC—not significant; Early BPMH vs. PPMC—significant.

^c^ Post hoc pairwise comparisons were performed using Mann–Whitney *U*‐tests with Bonferroni correction (*α* = 0.017) for total number of home medications: Usual care vs. Early BPMH—not significant; Usual care vs. PPMC—significant; Early BPMH vs. PPMC—significant.

### 3.2 Medication History and Reconciliation

The proportion of patients with one or more errors was 70.6% (T1), 50.8% (T2), and 5.8% (T3; *p* < 0.001) (Table 2). Medication reconciliation completion was 19.0% (T1), 26.2% (T2), and 98.1% (T3; *p* < 0.001). Time to BPMH was markedly shorter in T2 (2.6 h, IQR 1.6–4.4) and T3 (2.8 h, IQR 1.2–4.8) compared with T1 (26.7 h, IQR 21.2–64.4; *p* < 0.001), with no significant difference between T2 and T3.

**TABLE 2:** Primary outcome and medication errors stratified by type and risk.

Outcome	Study group			*p*
	T1‐usual care (*N* = 126)	T2‐early BPMH (*N* = 122)	T3‐PPMC (*N* = 103)	
Patient encounters ≥ 1 medication error, *n* (%)	89 (70.6%)	62 (50.8%)	6 (5.8%)	< 0.001^a^
Error type, % (*n*)				
Omitted	56.4% (154)	41.6% (99)	4.4% (6)	
Incorrect dose	5.9% (16)	1.7% (4)	1.5% (2)	
Incorrect route	1.1% (3)	4.2% (10)	0.0% (0)	
Incorrect or unnecessary drug	0.0% (0)	0.0% (0)	0.0% (0)	
Incorrect formulation	0.7% (2)	0.4% (1)	0.0% (0)	
Incorrect frequency	4.0% (11)	0.8% (2)	0.0% (0)	
Multiple error types^b^	2.6% (7)	2.1% (5)	0.0% (0)	
Risk level % (*n*)				
Low	23.1% (63)	27.7% (66)	3.6% (5)	
Moderate	10.6% (29)	6.7% (16)	0.7% (1)	
High	31.5% (86)	14.3% (34)	1.5% (2)	
Extreme	5.5% (15)	2.1% (5)	0.0% (0)	
Patients with a high or extreme risk error	39.7% (50)	23.8% (29)	1.9% (2)	

Abbreviations: BPMH, best possible medication history; PPMC, partnered pharmacist medication charting.

^a^ Post hoc pairwise comparisons for patients with one or more home medication errors were performed using the Marascuilo procedure and demonstrated significant differences between all groups.

^b^ Medication errors which could be classified as more than one type (e.g., dose and frequency or formulation and route).

### 3.3 Rate, Type and Risk Stratification of Errors

Medication omissions were the most frequent error type across all groups (Table 2). Incorrect dose was the next most common in T1 and T3; T2 had more incorrect route errors. No patient had an error involving an incorrect or unnecessary drug.

High‐ and extreme‐risk errors were markedly reduced with PPMC: 1.9% (T3, 95% CI 0.2–6.9) versus 39.7% (T1, 95% CI 31.2–48.2) and 23.8% (T2, 95% CI 16.3–31.4). The ARR for PPMC versus usual care was 0.65 (95% CI 0.558–0.742), with one error prevented per two patients (NNT = 2, 95% CI 2–2). For high or extreme risk errors, NNT = 3 (95% CI 3–4).

### 3.4 Secondary Outcomes

Secondary outcomes are summarised in Table 3. Median LOS was 3.3 days (T1, IQR 2.1–7.0), 1.6 days (T2, IQR 0.3–3.0), and 2.3 days (T3, IQR 1.1–8.0; *p* = 0.012). Dosing weight documentation during admission improved from 72.0% (T1) and 71.4% (T2) to 92.4% (T3; *p* < 0.001); pre‐administration entry improved from 49.3% and 50.0% to 83.3% (*p* < 0.001). Allergy status review during admission increased from 54.0% (T1) to 70.5% (T2) and 76.7% (T3; *p* < 0.001), though pre‐administration review did not differ significantly (43.2%, 51.6%, 53.4%; *p* = 0.219).

**TABLE 3:** Secondary outcomes.

Outcome	Study group			*p*
	Usual care (T1) (*N* = 126)	Early BPMH (T2) (*N* = 122)	PPMC (T3) (*N* = 103)	
Time from admission to BPMH (hours), median (IQR)	26.7 [21.2, 64.4]	2.6 [1.6, 4.4]	2.8 [1.2, 4.8]	0.001^a^
Total LOS (days), median (IQR)	3.3 [2.1, 7.0]	1.6 [0.3, 3.0]	2.3 [1.1, 8.0]	0.012^b^
MedRec completed, *n* (%)	24 (19.0%)	32 (26.2%)	101 (98.1%)	< 0.001^c^
Dosing weight^d^, *n* (%)				
Entered during admission	72.0% (54)	71.4% (70)	92.4% (61)	< 0.001^e^
Entered before a medication was administered	49.3% (37)	50.0% (49)	83.3% (55)	< 0.001^f^
Allergy status, *n* (%)				
Reviewed during admission	54.0% (68)	70.5% (86)	76.7% (79)	< 0.001^g^
Reviewed before a medication was administered	43.2% (54)	51.6% (63)	53.4% (55)	0.219

Abbreviations: BPMH, best possible medication history; IQR, interquartile range; MedRec, medication reconciliation; PPMC, partnered pharmacist medication charting.

^a^ Post hoc pairwise comparisons using Mann–Whitney *U*‐tests with Bonferroni correction (*α* = 0.017) demonstrated significantly longer time to BPMH in Usual care compared with Early BPMH and PPMC, with no significant difference between Early BPMH and PPMC.

^b^ LOS is presented descriptively for all groups. Comparative analyses were restricted to usual care and PPMC due to comparable haematology/oncology case‐mix, Mann–Whitney *U*‐test *p* = 0.012.

^c^ Post hoc pairwise comparisons for medication reconciliation completed during admission were performed using the Marascuilo procedure: Usual care vs. Early BPMH—not significant; Usual care vs. PPMC—significant; Early BPMH vs. PPMC—significant.

^d^ Dosing weight data excludes Haematology and Oncology patients (T1, *n* = 75; T2, *n* = 98; T3, *n* = 66).

^e^ Marascuilo pairwise comparisons for dosing weight completed during admission: Usual care vs. Early BPMH—not significant, Usual care vs. PPMC—significant, Early BPMH vs. PPMC—significant.

^f^ Marascuilo pairwise comparisons for dosing weight before a medication administered: Usual care vs. Early BPMH—not significant, Usual care vs. PPMC—significant, Early BPMH vs. PPMC—significant.

^g^ Marascuilo pairwise comparisons for allergy documented as reviewed during admission: Usual care vs. Early BPMH—significant, Usual care vs. PPMC—significant, Early BPMH vs. PPMC—not significant.

## 4 Discussion

PPMC was associated with substantially fewer medication errors and a marked reduction in patients experiencing high‐ and extreme‐risk errors compared with both early BPMH and usual care, the first such evaluation in a paediatric eMMS setting to our knowledge. The ARR was 0.65 (95% CI 0.558–0.742), with one error prevented per two patients (NNT = 2) and one high or extreme risk error prevented per three (NNT = 3). These findings are consistent with adult PPMC literature: the Victorian rural/regional cohort reported NNT = 1.75 (95% CI 1.63–1.89) for any error [14], and the Tasmanian ED study NNT = 4.0 for high or extreme risk errors [15]. Consistency across adult and paediatric populations, and across paper‐based and electronic environments, supports PPMC as a generalisable model of care.

In eMMS, transcription errors are eliminated, so early BPMH would be expected to outperform paper‐based settings. Despite this, our T2 error rate (50.8%) closely mirrored the Tasmanian finding (49.4%) [15], while PPMC reduced errors further to 5.8%, demonstrating that PPMC's collaborative decision‐making and pharmacist charting provide meaningful benefit beyond early BPMH even within eMMS.

Medication omissions were the most common error type, consistent with prior PPMC studies. Dosing errors predominated in usual care and PPMC, whereas early BPMH had more route‐related errors, usually involving PEG/PEJ administration being incorrectly charted for oral administration, likely reflecting that doctors charting without pharmacist input had less familiarity with enteral route requirements. High‐severity errors in this paediatric cohort involved haematology/oncology patients, with extreme‐risk errors including corticosteroid and mercaptopurine omissions and a 100‐fold tacrolimus overdose, consistent with oncology literature [13]. Supportive care omissions were similarly rated high risk.

LOS was shorter in the PPMC group, though causality cannot be inferred from this retrospective design. LOS is influenced by diagnosis mix, illness complexity, bed availability, and ED overcrowding. Given comparable haematology/oncology representation, inferential LOS analysis was restricted to T1 versus T3; T2 LOS is presented descriptively only. PPMC significantly improved medication reconciliation (98.1% vs. 19.0% in T1), dosing weight documentation, and allergy review, reflecting the structured and reliable nature of pharmacist‐led charting. Medication reconciliation completion remained low in the early BPMH group (26.2%), despite a BPMH being available within 4.4 h for 75% of patients. This suggests that availability of accurate medication information alone is insufficient to drive reconciliation documentation; the act of reconciliation requires deliberate, structured completion, which the PPMC model embeds by design through the pharmacist's direct involvement in charting. This finding aligns with broader literature demonstrating that passive information transfer at transitions of care rarely translates into reliable documentation without an active accountable process [17]. Allergy status review showed improvements across both early BPMH and PPMC groups; however, many patients had allergy information documented in progress notes rather than the designated eMR allergy field, with consistent use of the structured field most evident in the PPMC group.

### 4.1 Strengths and Limitations

A key strength was the real‐world evaluation of PPMC shortly after implementation, providing insight into safety outcomes for newly credentialed pharmacists and extending prior adult methods to a paediatric eMMS setting.

Limitations include the non‐randomised design. Prospective randomisation was ethically precluded given established adult PPMC evidence and heightened paediatric vulnerability. A matched retrospective cohort was infeasible given small, unequal group sizes; random sampling was not pursued as between‐group case mix differences, particularly haematology/oncology distribution, would have persisted. Future prospective studies with concurrent control arms or interrupted time series designs are recommended. To address potential confounding from the unequal haematology/oncology distribution, a sensitivity analysis was completed excluding haematology/oncology patients. The proportion of patients with one or more medication errors was 66% in usual care (T1, *n* = 75), 51% in early BPMH (T2, *n* = 98), and 6.1% in PPMC (T3, *n* = 66) (*p* < 0.001), consistent with the primary analysis and confirming that differences in haematology/oncology representation between groups did not materially influence the primary outcome.

Only patients who had a BPMH from a pharmacist were included in this research. This criterion ensured comparability with the PPMC model of care, where a pharmacist‐led BPMH is a mandatory component, and allowed consistent identification of medication errors on admission, particularly omission errors. This study did not collect data on actual patient harm resulting from medication errors, nor did it distinguish between errors intercepted before reaching the patient and those that were not. Only the potential for harm was assessed. As actual harm and interception status were not measured as part of the study design, this represents a gap in the dataset, and the reported error and risk rates should be interpreted as indicators of potential rather than realised harm.

Expert panel assessment did not formally distinguish intentional from unintentional discrepancies, potentially resulting in modest overestimation of error prevalence, consistent with prior Victorian PPMC methodology. Secular trends, including introduction of an ED pharmacist in 2023 and increased pharmacy staffing, may have independently influenced outcomes, though the magnitude of T2‐to‐T3 improvement suggests PPMC contributed beyond background trends.

## 5 Conclusion

This study demonstrates that PPMC substantially reduces medication errors for children and young people presenting to hospital via the ED, preventing one error per two patients and one high or extreme risk error per three. The benefit was not explained by access to an accurate medication history alone. Early BPMH improved error rates but fell well short of PPMC, underscoring that PPMC creates an accountable process that passive information transfer cannot replicate. Improvements in medication reconciliation, dosing weight documentation, and allergy review reinforce this, with reconciliation remaining poor even when doctors had a pharmacist‐completed history available to them. These findings extend a well‐established adult model into a paediatric electronic prescribing environment for the first time, and the consistency of effect sizes across settings strengthens the case for broader implementation. Children admitted to hospital are at heightened risk of preventable medication harm and PPMC offers a practical and scalable response.

## Funding

The authors have nothing to report.

## Ethics Statement

Ethics approval was granted by the Sydney Children's Hospital Network Human Research Ethics Committee (Reference: 2024/ETH01235).

## Conflicts of Interest

The authors declare no conflicts of interest.

## Supporting information


**Data S1:** STROBE checklist: cohort studies.

## Data Availability

Research data are not shared.
