# Supplementary material for: Partnered Pharmacist Medication Charting for Children and Young People: A Retrospective Cohort Study
Source: J Paediatr Child Health. 2026 Jul 28;62(9):1723–9. doi: 10.1111/jpc.70505 (PMC13617220; doi:10.1111/jpc.70505)
Supplement: Supplementary file 1 [file JPC-62-1723-s001.docx]

**STROBE CHECKLIST: COHORT STUDIES**

*Manuscript: Partnered Pharmacist Medication Charting in Children and Young People*

| **Item** | **STROBE Recommendation** | **Manuscript Location** |
| --- | --- | --- |
| 1a | Indicate study design in title/abstract | Abstract – Methods (retrospective cohort study); study design not stated in title |
| 1b | Balanced summary of methods and findings | Abstract – Results & Conclusions |
| 2 | Scientific background and rationale | Introduction (entire section) |
| 3 | Specific objectives/hypotheses | End of Introduction (study aims and objectives) |
| 4 | Key elements of study design | Methods (opening paragraphs; T1–T3 time periods; inclusion/exclusion criteria) |
| 5 | Setting, location, dates, data periods | Methods – Time periods T1–T3; ED of a tertiary paediatric hospital |
| 6a | Eligibility criteria and selection methods | Methods – Inclusion and exclusion criteria |
| 6b | Matching criteria (if applicable) | Not applicable (no matching used) |
| 7 | Definitions of outcomes, exposures, confounders | Methods – Primary and secondary outcomes; error classification |
| 8 | Data sources and measurement methods | Methods – BPMH, eMR review, expert panel, SHPA risk tool |
| 9 | Potential bias and how addressed | Discussion – Strengths and Limitations |
| 10 | How study size was determined | Not reported (retrospective sample of eligible encounters) |
| 11 | Handling of quantitative variables | Methods – Statistical analysis |
| 12a | Statistical methods including confounding | Methods – Statistical analysis |
| 12b | Subgroup or interaction analyses | Methods – Post-hoc Marascuilo comparisons |
| 12c | Handling of missing data | Methods – Missing data approach described. Absence of eMR documentation for reconciliation, dosing weight, and allergy fields was treated as a negative outcome rather than missing data, reflecting a clinically meaningful failure of documentation. Cases were excluded only where source documentation was inaccessible. See also item 14b. |
| 12d | Loss to follow-up | Not applicable (retrospective cohort) |
| 12e | Sensitivity analyses | Discussion – Strengths and Limitations. Sensitivity analysis performed excluding haematology/oncology patients from the primary outcome analysis. Results were consistent with the primary analysis: proportion of patients with one or more medication errors was 68.1% (T1, n=72), 43.8% (T2, n=96), and 7.0% (T3, n=57) (p<0.001), confirming that differences in haematology/oncology representation between groups did not materially influence the primary outcome. |
| 13a | Numbers at each study stage | Results – Patient Characteristics (text); Supplementary flow diagram |
| 13b | Reasons for non-participation | Results – Exclusions and reasons |
| 13c | Flow diagram considered | Supplementary STROBE flow diagram (to be uploaded) |
| 14a | Participant characteristics | Results – Table 1 |
| 14b | Number with missing data | Minimal missing data. Non-documented medication reconciliation, allergy review and dosing weight fields were analysed as study outcomes rather than treated as missing data. Cases with unavailable source documentation (no Best Possible Medication History) were excluded prior to analysis. |
| 14c | Follow-up summary | Not applicable (no follow-up period) |
| 15 | Outcome events over time | Results – Tables 2 and 3 (outcomes by group T1–T3) |
| 16a | Unadjusted and adjusted estimates (95% CI) | Results – Main analysis (unadjusted estimates only) |
| 16b | Category boundaries | Results – Error risk stratification (SHPA 1–5 categories) |
| 16c | Absolute risk estimates | Results – ARR and NNT reported |
| 17 | Other analyses (subgroups/sensitivity) | Results – Secondary outcomes (LOS, reconciliation, dosing weight, allergy) |
| 18 | Summary of key results | Discussion – Summary of findings |
| 19 | Limitations and bias | Discussion – Strengths and Limitations |
| 20 | Overall interpretation | Discussion – Interpretation |
| 21 | Generalisability | Discussion – Strengths and Limitations |
| 22 | Funding sources and role | Funding statement in manuscript: 'This research received no specific grant from any funding agency in the public, commercial, or not-for-profit sector.' |
